# Aseptic Abscesses and Inflammatory Bowel Disease: Two Cases and Review of Literature

**DOI:** 10.1155/2017/5124354

**Published:** 2017-02-06

**Authors:** Natasha Bollegala, Rishad Khan, Michael A. Scaffidi, Ahmed Al-Mazroui, Jenna Tessolini, Adrienne Showler, Errol Colak, Samir C. Grover

**Affiliations:** ^1^Division of Gastroenterology, Women's College Hospital, Toronto, ON, Canada; ^2^Division of Gastroenterology, St. Michael's Hospital, Toronto, ON, Canada; ^3^Division of Infectious Disease, St. Michael's Hospital, Toronto, ON, Canada; ^4^Department of Medical Imaging, St. Michael's Hospital, Toronto, ON, Canada

## Abstract

*Background*. Aseptic abscesses (AA) are sterile lesions that represent an extraintestinal manifestation (EIM) of inflammatory bowel disease (IBD). Though Canada has the highest prevalence of IBD in the world, reports of IBD-associated AA are absent in Canada. This may represent a different IBD phenotype or underrecognition and underreporting.* Purpose*. To explore AA as a possible EIM of IBD and evaluate clinical and investigative findings among patients with IBD-associated AA.* Methods*. Retrospective chart and literature reviews were performed to find cases of IBD-associated AA at our institution and in the literature.* Results*. We identified 2 cases of IBD-associated AA in our institution. Both patients had ulcerative colitis and presented with fever, abdominal pain, and weight loss. Radiological workup and aspiration showed sterile splenic abscesses. The AA were unresponsive to antibiotics. One patient improved on corticosteroids and one underwent splenectomy. We retrieved 37 cases of IBD-associated AA from the literature. All patients showed no evidence of infection, failed to resolve with antibiotics, and, if attempted, improved on corticosteroids.* Conclusions*. Our cases are the first reported in Canada. They support literature which suggests AA as an EIM of IBD and may help increase recognition and reporting of this phenomenon.

## 1. Introduction

Aseptic abscesses (AA) are a rare and serious extraintestinal manifestation (EIM) of inflammatory bowel disease (IBD). Despite having the highest incidence and prevalence of IBD in the world, there are no reports of this entity in Canada [1–4]. In this study, we describe two local cases and review the literature on AA with underlying IBD.

IBD-associated AA is a rare condition with fewer than 50 cases reported, largely in the European literature [5]. AA are deep abscesses with neutrophilic features associated with negative cultures and serologic tests, failure of antibiotic therapy, and improvement on corticosteroids. Due to the scarcity of reported cases and absence of observational and experimental studies, clinicians face challenges in diagnosing and managing patients. This report evaluates two cases of IBD-associated AA for clinical and investigative findings to add to the sparse Canadian literature.

## 2. Methods

### 2.1. Chart Review

This retrospective single center study was approved by our Institutional Review Board with a waiver for informed consent. Our radiology information system (syngo, Siemens Medical Solutions USA, Inc., Malvern, PA) was searched using Montage Search and Analytics (Montage Healthcare Solutions, Philadelphia, PA) for CT examinations performed between January 2005 and July 2016. The terms “splenic abscess,” “aseptic abscess,” “inflammatory bowel disease,” “ulcerative colitis,” and “Crohn's disease” were used. The Soarian Clinicals database (Cerner Corporation, Kansas City, MO) was used to find additional clinical data on identified patients. Diagnoses of AA were made based on (1) history of IBD; (2) imaging of focal enclosed lesions with the typical appearance of abscesses; (3) failure of antibiotic therapy; and (4) negative blood and aspirate. Patient charts were analyzed for demographics, symptoms, medical comorbidities, disease diagnosis and progression, and management.

### 2.2. Literature Review

For the literature review, an electronic search of the online databases MEDLINE, EMBASE, and CINAHL as well as the reference lists of retrieved articles was performed to identify potential articles published in print or online before July 14, 2016. Search terms included “Inflammatory bowel disease”, “aseptic splenic abscess”, “aseptic abscess”, and “extraintestinal”. Abstracts of all publications found through the above search strategy were screened for relevancy. Full-text articles, if available, were retrieved. If a translation for a non-English article was not available, authors were contacted for full-text translations. If they did not respond, these articles were excluded from the literature review.

## 3. Results

### 3.1. Case Reports

We describe two cases of IBD-associated AA of patients who presented to St. Michael's Hospital in Toronto. The clinical characteristics of our two cases are summarized in Table 1.

#### 3.1.1. Case One

A 33-year-old man with a history of ulcerative colitis (UC) and prior subtotal colectomy and ileal pouch-anal anastomosis (IPAA) presented to hospital with a six-day history of fevers, nonradiating left-upper quadrant abdominal pain worse with movement, a 15 lbs weight loss, and genital skin lesions with the typical appearance of pyoderma gangrenosum. His bowel movements were unchanged from 8–10 nonbloody stools per day. Past medical history was significant for corticosteroid-refractory pancolonic UC managed with subtotal colectomy and IPAA at age 18. Surgical pathology on the colectomy specimen confirmed ulcerative pancolitis. In the subsequent 15 years, he was treated for three episodes of pouchitis where each responded well to three weeks of ciprofloxacin and metronidazole

**Figure 1:**
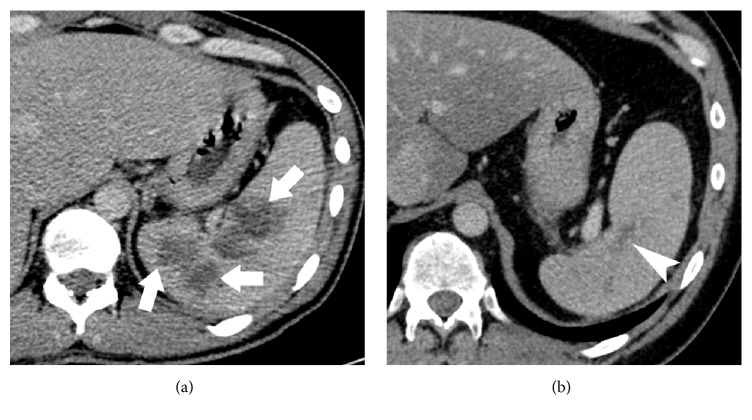
Axial contrast enhanced CT images demonstrating (a) multiple splenic abscess (thick arrows) with (b) resolution and residual scarring (thin arrow) following treatment with steroids.

On presentation, laboratory testing showed an elevated white blood cell (WBC) count of 18.0 × 10^3^ cells/*μ*L with a neutrophil count of 16.4 × 10^3^ cells/*μ*L, C-reactive protein (CRP) of 114.9 mg/L, and erythrocyte sedimentation rate (ESR) of 51 mm/hr. An abdominal CT scan revealed multiple mostly confluent hypodense lesions in the spleen, the largest being 7.0 × 3.2 × 5.2 cm (Figure 1(a)), concerning for abscesses. Aspiration of the largest abscess revealed pus. The abscesses were drained and he was started on ceftriaxone and metronidazole.

The patient failed to improve. Cultures of blood and the aspirate remained sterile even after 7-day incubation. All antibiotics were then stopped. Pouchoscopy revealed inflammation in the IPAA with no inflammation in the pouch or proximal small bowel. A repeat abdominal CT scan showed progression of the splenic lesions. The patient was started on IV corticosteroids. His abdominal pain, fevers, and genital skin lesions improved and he was discharged home on a tapering course of oral steroids and rectal aminosalicylates. He was admitted one year later with a one-month history of left-sided flank pain, sweats, fevers, and abdominal pain with no change in bowel habits or weight loss. An abdominal and pelvic CT scan showed multiple focal ill-defined hypoattenuating lesions scattered throughout the spleen with the largest measuring 4.2 cm in diameter. Aspiration of an abscess proved sterile. Pouchoscopy showed active inflammation.

The patient was commenced on IV steroids and transitioned to an oral tapered dose of prednisone. Additionally, he was initiated on 5 mg/kg of infliximab every eight weeks, increased to 10 mg/kg due to lack of response. His abdominal pain and fever ceased and a repeat abdominal CT scan six months later revealed resolution of the multiple splenic abscesses (Figure 1(b)).

#### 3.1.2. Case Two

**Figure 2:**
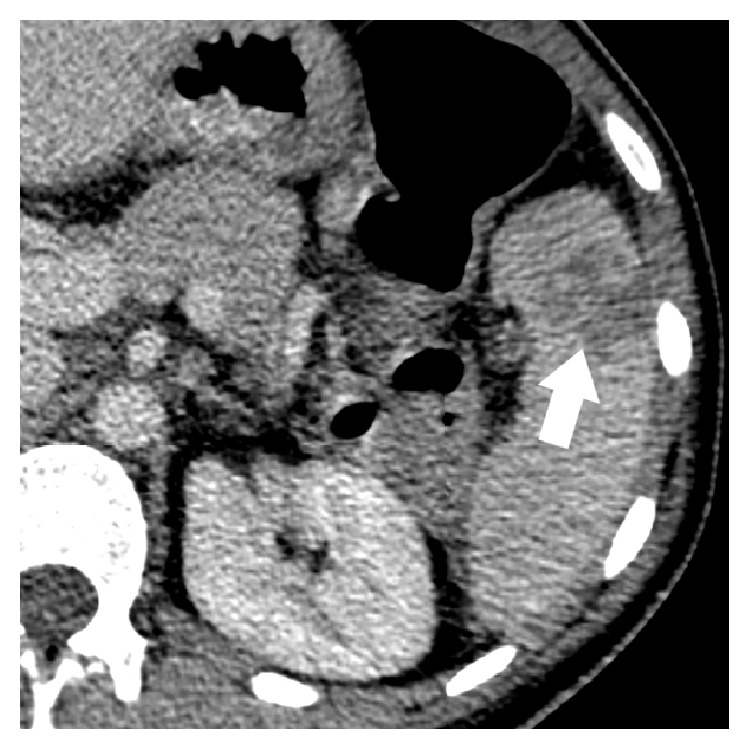
Axial contrast enhanced CT image demonstrating a heterogeneous splenic abscess (thick arrow).

A 27-year-old woman presented to hospital with fever, abdominal pain, chest pain, and weight loss. Past medical history was of UC diagnosed at age 26, treated with prednisone and azathioprine, for which she was nonadherent and ceased therapy. At the time of presentation, she described four days of left-upper quadrant abdominal pain and pleuritic chest pain radiating to her left shoulder. There was fever, oral ulcers, a 10 lbs weight loss, and arthritis of the hands, elbows, knees, and ankles. She had an elevated WBC count (21.1 × 10^3^ cells/*μ*L), neutrophil count (16.0 × 10^3^ cells/*μ*L), CRP (112 mg/L), and ESR (88 mm/hr). Abdominal CT scans revealed an enlarging heterogeneous abscess measuring 3.7 × 4.0 × 2.4 cm initially and 4.2 × 3.6 × 2.6 cm 3 days later (Figure 2).

Aspiration of the splenic collections revealed pus. Cultures of blood and aspirate were negative. The patient declined further endoscopic staging of her UC. She underwent splenectomy and the resected spleen contained multiple aseptic abscesses. Histology revealed areas of neutrophil-dominant necrotic tissue.

The patient presented two months later with fever, arthralgia, oral ulcers, and tender papules on her buttocks, arms, and legs. Biopsy of the lesions revealed neutrophilic dermatosis (ND). Based on her clinical and histological findings, she was diagnosed with Sweet's syndrome and started on prednisone 60 mg PO daily. Her dermatologic and rheumatologic symptoms rapidly resolved. She was initiated and remains on infliximab 5 mg/kg every six weeks, continuing to do well on this regimen.

### 3.2. Literature Review of Aseptic Abscess in Inflammatory Bowel Disease

A total of 37 cases of IBD-associated AA were found in our literature search of MEDLINE, EMBASE, and CINAHL and through hand-searching. These articles were published from 1994 [6] to 2016 [20, 21]. The clinical characteristics of these cases can be found in Table 1. Of the 37 total patients, approximately 65% (*n* = 24) were female and 35% (*n* = 13) were male. The mean ages of patients with AA and IBD at the time of diagnosis were 28.9 years (range 10–80 years) and 26.1 years (range 12–72 years), respectively.

IBD preceded the diagnosis of AA in 48.6% of patients (*n* = 18), was concomitant in 24.3% (*n* = 9), and appeared subsequently in 27.0% (*n* = 10). With respect to IBD type, 73.0% of patients had underlying CD (*n* = 27), 24.3% (*n* = 9) had UC, and 2.7% (*n* = 1) had indeterminate colitis. During their diagnosis of AA, 45.9% patients (*n* = 17) had an IBD flare, while 54.1% (*n* = 20) did not. Regarding initial symptoms, 83.8% of patients presented with fever (*n* = 31), 67.6% with abdominal pain (*n* = 25), 32.4% with diarrhea (*n* = 12), and 32.4% with weight loss (*n* = 12). The most common AA locations were the spleen (*n* = 23) and liver (*n* = 5), with 32.4% of patients found to have lymph node involvement (*n* = 12). 10.8% of patients had ND (*n* = 4).

Regarding treatment, 91.9% of patients received antibiotics (*n* = 34), to which they were all unresponsive. Conversely, 94.6% received and responded to corticosteroid therapy (*n* = 35). 40.5% of patients (*n* = 15) received additional immunosuppressive therapy of azathioprine (*n* = 12), cyclophosphamide (*n* = 3), methotrexate (*n* = 1), infliximab (*n* = 2), and adalimumab (*n* = 1). Additionally, 37.8% of patients underwent splenectomy (*n* = 14), 13.5% had incision and drainage of abscesses (*n* = 5), and 5.4% had lymph node excision (*n* = 2). Relapses were reported in 54.1% of patients (*n* = 20), with the average number of relapses among all 37 cases being 1.22 (range: 0–5). The implementation (or absence) of maintenance therapy was reported in 45.9% of cases (*n* = 17), with 47.1% of these patients placed on maintenance therapy (*n* = 8/17), including low-dose prednisone (*n* = 3), azathioprine (*n* = 4), sulfasalazine (*n* = 3), and adalimumab (*n* = 1). Among the patients receiving maintenance therapy, 5 relapses occurred while on prednisone maintenance therapy. Alternatively, no relapses occurred while patients were on azathioprine, sulfasalazine, or adalimumab.

## 4. Discussion

We present two cases of aseptic abscesses, a rare EIM of IBD. Our patients were aged 33 and 27 years old, respectively, and both had underlying UC. Both patients presented with fever, abdominal pain, and weight loss and had splenic lesions which proved sterile. Patient 1 did not respond to antibiotics, improved on corticosteroids, and was maintained with infliximab. Patient 2 received a splenectomy and subsequently was maintained with infliximab.

### 4.1. Clinical Diagnosis

There are no accepted guidelines for the diagnosis of AA. However, Andre and colleagues created a set of common characteristics based on a case series with literature review: (1) deep abscesses with neutrophilic features; (2) negative serologic tests for bacteria and fungi and cultures of blood and aspirate; (3) when administered, failure of broad-spectrum antibiotic therapy including antituberculosis therapy; (4) rapid clinical improvement on corticosteroid therapy with or without additional immunosuppressant therapy and subsequent radiologic evidence of abscess resolution [5]. Our patients were assessed using this definition of AA, with patient 1 meeting all criteria and patient 2 meeting all but the fourth criterion (i.e., corticosteroid therapy), as corticosteroids were not attempted on this patient. Additionally, when applying these criteria to patients from the literature, we found multiple reports of superficial AA in IBD. Sterile abscesses were reported on the upper eyelids, scalp, face, chest, forearm, and nasal septum [8, 10, 11, 13, 18]. These findings may warrant expansion of criterion 1 to include superficial AA.

### 4.2. Pathology

There is some ambiguity on how to best describe sterile lesions with underlying IBD. Superficial lesions have been described as neutrophil-dominant with surrounding granulomatous tissue [10, 11, 13]. Similarly, in our report, aspiration of patient 2's splenic abscess revealed areas of neutrophil-dominant necrosis. Lesions in previously published cases have also been described as rheumatoid nodules, though this assertion has been debated [12, 22]. With this lack of consensus in mind, the most accurate term to describe these lesions is “aseptic abscess” [5].

AA shares a similar pathological picture with ND, showing polymorphonuclear leukocyte infiltrates and sterile abscesses [23–25]. Both of our patients had ND: patient 1 developed pyoderma gangrenosum, and patient 2 developed Sweet's syndrome. In the literature, 10.8% of patients had ND (*n* = 4). Sweet's syndrome and pyoderma gangrenosum are recognized complications of IBD, though it is unclear if their incidence is higher in IBD patients with AA compared to those without AA [23, 25].

### 4.3. Association with Inflammatory Bowel Disease

AA has been documented as an EIM found before, during, and after the diagnosis of IBD [5]. Our patients had established IBD diagnoses before any clinical symptoms of AA appeared. Other cases in our institution may not have been found through the database search if the AA preceded IBD and thus was not classified as an EIM. This may indicate a need to perform colonoscopy on patients with AA and no underlying IBD, as 51.3% of published cases reported IBD diagnoses concomitant with or following AA diagnoses (*n* = 19).

With regard to types of IBD, both of our patients had UC compared to only 24.3% (*n* = 9) of patients reported in the literature. A recent case report by Boschetti et al. of AA described a diagnosis of multiple systemic abscess syndrome, though they called this a complication of CD and not IBD, removing patients with UC from consideration [21]. Despite the higher incidence of CD, compared to UC-associated AA, it is important for clinicians to realize that AA can be an EIM regardless of the type of underlying IBD.

The association between disease severity of IBD and the manifestation of AA is unclear. Among included studies, only one reported clinical or endoscopic measures of disease activity for IBD, such as the Crohn's Disease Activity Index (CDAI) or the Crohn's Disease Endoscopic Index of Severity (CDEIS) [11, 26, 27]. Though inflammatory biomarkers such as CRP and ESR were commonly presented, they cannot be used as surrogates IBD activity, as AA is an inflammatory process itself.

### 4.4. Treatment

AA has been treated with corticosteroid therapy, azathioprine, cyclophosphamide, methotrexate, infliximab, adalimumab, incision and drainage, and splenectomy. From the literature and from our own institution, there were 36 of 39 patients that received and responded to corticosteroid therapy, which indicates its effectiveness as the first-line for induction. Maintenance therapy should follow because relapse is common, as seen in over half of the patients in the literature review and in one of our two patients. Interestingly, no patients in the literature experienced relapse while on maintenance therapy of azathioprine, sulfasalazine, adalimumab, or infliximab, although the durability of this response is unknown [7, 8, 10, 13, 17, 19, 21]. There were no reported fatal outcomes.

## 5. Conclusions

The cases described in this study are the first to be reported in Canada. They highlight the importance of recognizing IBD-associated AA in at-risk patients. The lack of diagnostic guidelines can lead clinicians to forego potentially effective corticosteroid and immunosuppressant therapy in favour of surgical intervention, as in our patient 2 who received a splenectomy. Future research should be aimed towards rigorous multicentre prospective case-control studies with greater participant numbers to better understand the natural history of this condition, to form the foundation for diagnostic criteria and to help improve recognition, reporting, and management.

## Figures and Tables

**Table 1:** Summary of IBD associated AA cases from the literature database and our search at St. Michael's Hospital.

Study/patient ID	Age/sex	IBD phenotype (CD/UC/IC)	Age of IBD diagnosis/temporal relation to diagnosis of AA	IBD flare during AA	Symptoms	Location of AA	Other IBD EIM	Antibiotic treatment	Corticosteroids	Additional immunotherapy	Surgical procedures	Maintenance therapy after diagnosis of AA	Number of relapses
André et al. 2007 [5]^*∗*^/1–21	Mean 24.4 (range 10–54)/12 F & 9 M	17CD, 3UC, 1IC	Average age of IBD onset = 23.7 (range 12–38)/before (*n* = 7), concomitant (*n* = 7), after (*n* = 7)	Yes (*n* = 8), no (*n* = 13)	Fever (*n* = 19), abdominal pain (*n* = 17), weight loss (*n* = 10), diarrhea (*n* = 5)	Spleen alone (*n* = 6) or spleen and lymph nodes (*n* = 4); pharynx and cervical muscles (*n* = 1); liver (*n* = 1); liver and lymph nodes (*n* = 3); kidney (*n* = 1), and lymph nodes alone (*n* = 4)	Arthritis (x4), myalgia (x5), ND (x2), aphthous ulcer (x7)	Yes (*n* = 21)	Yes (*n* = 21)	Total (*n* = 10), cyclophosphamide (*n* = 3), azathioprine (*n* = 7), methotrexate (*n* = 1), infliximab (*n* = 2)	Splenectomy (*n* = 9)	Information not provided	Mean = 1.38 (*n* = 12), range 0–5

Lamport et al. [6]/22	38/F	CD	34/before	No	Leg weakness	Bilateral psoas muscle and epidural	—	Yes	Yes	—	—	—	1

Actis et al. [7]/23	30/M	CD	30/after	Yes	Fever, diarrhea, abdominal pain	Spleen, abdominal lymph nodes	Panniculitis, polyneuropathy	Yes	Yes	Azathioprine	Splenectomy and lymph node excision	Prednisone (stopped after 4th relapse), azathioprine (after last relapse)	4

Tirpitz et al. [8]/24	80/F	UC	72/before	Yes	Abdominal pain, abdominal swelling, bloody diarrhea	Bi-temporal upper eyelids	Pyoderma gangrenosum	Yes	Yes	—	—	Prednisone, sulfasalazine	0

Murata et al. [9]/25	18/F	UC	15/before	No	Fever	Sternum	Arthritis	Yes	Yes	—	I&D	—	4

Hara et al. [10]/26	39/M	UC	34/before	No	Painful facial lesions, fever, nonbloody diarrhea	SC scalp, face, right inner canthus, submaxilla, and chest	Arthritis	Yes	Yes	—	I&D	Sulfasalazine (after relapse)	1

Coat et al. [11]/27	31/F	CD	28/before	Yes	Fever, back pain	Spleen	—	Yes	Yes	Azathioprine	Splenectomy	—	1

Kinjo et al. [12]/28	34/F	UC	31/before	Yes	Fever, bloody stool	SC sternum	Arthritis	No	Yes	—	I&D	—	1

Holstein et al. [13]/29	26/M	CD	24/before	No	Fever, diarrhea, weight loss, weakness, loss of appetite	Liver, spleen	HLA B27 sacroiliitis, Arthritis	Yes	Yes	Azathioprine	Splenectomy	Azathioprine (after 3rd relapse)	3

Li et al. [14]/30	39/F	UC	26/before	Yes	Fever, bloody diarrhea, abdominal pain	SC left forearm	Arthritis	Yes	Yes	—	I&D	—	0

Coyne [15]/31	34/M	CD	33/before	No	Abdominal pain	Spleen	—	No	No	—	Splenectomy	—	0

Renna et al. [16]/32	20/F	CD	20/concomitant	Yes	Fever, abdominal pain, nonbloody diarrhea, weight loss	Spleen	—	Yes	Yes	—	Splenectomy	Prednisone (before, during, and after relapse)	1

Zakout et al. [17]/33	29/F	CD	29/concomitant	Yes	Fever, abdominal pain, pain in right shoulder radiating from abdomen, right pleuritic chest pain, nonbloody diarrhea	Liver	—	Yes	No	Azathioprine	—	Azathioprine, sulfasalazine	0

Yilmaz et al. [18]/34	34/F	UC	22/before	Yes	Fever, nasal ache, difficulty with breathing	Nasal septum	—	Yes	Yes	—	I&D	—	0

Brooks and Ghaffari [19]/35	19/F	CD	19/concomitant	Yes	Abdominal pain	Spleen	Pustular skin lesions	Yes	Yes	Azathioprine	—	Azathioprine	0

Sakharpe et al. [20]/36	48/F	CD	Unknown/before	No	Fever, weakness, loss of appetite, coughing, chest pain	Liver	—	No	Yes	—	—	—	0

Boschetti et al. [21]/37	40/F	CD	Unknown/after	No	Fever, abdominal pain	Spleen, pancreas	Sweet's syndrome	Yes	Yes	Adalimumab	Laparoscopic biopsy of mesenteric lymph nodes	Adalimumab	0

Bollegala et al./case 1	33/M	UC	18/before	Yes	Abdominal pain, fever, weight loss, sweat	Spleen	Pyoderma gangrenosum	Yes	Yes	Infliximab	—	Prednisone & 5-ASA (before relapse), infliximab (after relapse)	1

Bollegala et al./case 2	27/F	UC	26/before	No	Abdominal pain, chest pain radiating to left shoulder, fever, weight loss	Spleen	Sweet's syndrome, oral ulcers, arthritis	No	No	Infliximab	Splenectomy	Infliximab	0

AA = aseptic abscess, CD = Crohn's disease, EIM = extraintestinal manifestation, F = female, IC = indeterminate colitis, IBD = inflammatory bowel disease, I&D = incision and drainage, M = male, ND = neutrophilic dermatosis, SC = subcutaneous, UC = ulcerative colitis.

^*∗*^Case series summary data available.

## Abbreviations

AA: Aseptic abscesses
EIM: Extraintestinal manifestation
IBD: Inflammatory bowel disease
CD: Crohn's disease
UC: Ulcerative colitis
IC: Indeterminate colitis
IV: Intravenous
WBC: White blood cell
ND: Neutrophilic dermatoses.
